# Implicated by scale: Anthropochemicals and the experience of ecology

**DOI:** 10.1177/00380261221084780

**Published:** 2022-03-31

**Authors:** Dimitris Papadopoulos

**Affiliations:** Institute for Science and Society, School of Sociology and Social Policy, University of Nottingham, UK

**Keywords:** chemical pollution, ecological experience, limits of growth, material milieu, production scale, reparation ecology, science and technology

## Abstract

If our worlds are unimaginable, or, ironically, perhaps even unsustainable without anthropogenic chemicals, what does it mean to live and navigate the toxic regime, this historical moment where human-made substances are so entangled with ecologies and societies that a clean up and an ‘after’ to our polluted worlds is almost unthinkable? Anthropogenic chemicals are produced and used at such scale that humans need a tremendous scale of alternative chemicals to replace them. Scale, the organising principle of growth, is the source of ecological degradation and, simultaneously, is a necessary component of many remediation attempts. As life is becoming more and more chemical, chemical practice is gradually becoming conscious of its flagrant disregard of its own ecological boundaries. The attempt to restore a holistic experience of ecology shapes many current attempts to develop alternative chemical practices. When chemical practice becomes obliged by ecology to respond to the environmental crisis, the search for a different approach to scale emerges. With obligation comes the quest for reparation, both as repair and as compensation for the social and ecological damage done.

## Being implicated: Anthropochemicals

In the print *Peace Through Chemistry* (1970), Roy Lichtenstein depicts a tree branch along test tubes and microscopes to mix cubism, art moderne, pop art and the scale of muralism into a promise and a vision for science’s contribution to peace. Peace is something that very few would associate with chemistry, even in the 1970s where the creation of new synthetic compounds and materials corroborated the rise of consumerism and the plastic culture. Lichtenstein’s *Peace Through Chemistry* feels more in line with the extensive and carefully planned public relations, press and international diplomacy campaign of the US government to ease people’s and allies’ Cold War anxieties (Orr, 2006) and simultaneously to prepare the build-up of the US nuclear weapons arsenal – a campaign that was marked by President Eisenhower’s UN General Assembly speech *Atoms for Peace* in 1953. One wonders if Lichtenstein was ironical in his apotheosis of science and in elevating chemistry to a guarantor of peace just eight years after the publication of Rachel Carson’s *Silent Spring* (1962) and in a historical moment where the US military was deploying extensive chemical warfare in Vietnam and was spraying Agent Orange across Southeast Asia to destroy forests, crops, livelihoods and people. As much as the promise of chemistry might be detached from the reality of chemical substances, in today’s environmental conditions this statement appears more pertinent than ever; it becomes in fact an imperative for action and an imperative for transformation.

We can turn the title of this series of lithographs and say that if there is no peace through chemistry then there will not be any. From plastics to endocrine and hormone disrupting compounds, from the depletion of rare earth metals to persistent environmental pollutants, from the multiplication of public health hazards to the decline of food pollinating insects, anthropogenic chemicals pose a vital challenge to social and ecological worlds. The embeddedness of human-made chemicals in modern societies and in industry is so deep that we can talk of the anthropogenic chemicalisation of the social and natural world (Barry, 2005; Bensaude-Vincent & Stengers, 1996; Fortun, 2014). Anthropochemicals, all these human-made synthetic substances that reign over our everyday lives and environments, have become the most visible and ubiquitous marker of industrial humanity as a geological agent (Masco, 2021) investigated by the Working Group on the Anthropocene (Zalasiewicz et al., 2011, 2016). Fewer than 10% of all human-made chemicals are environmentally, socially or clinically benign, leading to detrimental problems on a planetary scale (Boudia et al., 2018; Sanderson & Anastas, 2011). Anthropochemicals protract life for some and administer death to others; they secure life and they let die. In contemporary Global North societies, the production of life truly relies on chemicals. More than 95% of all manufactured products contain some form of synthetic chemicals. Life and death is governed through anthropochemicals.

Anthropochemicals reveal the differential effect that the sourcing, production, use or afterlife of manufactured chemicals has on certain social groups and on certain places and its human and nonhuman inhabitants rather than other. It is to say that securing life today for some populations cannot happen without letting other populations die. There is a planetary system of production, circulation, application and disposal of chemicals that imposes the effects of toxicity and pollution more on certain places and social segments than others (Bullard, 1994; Davies, 2018; Nixon, 2011). Race, class and geographical location are vectors that navigate how environmental damage is attached only to certain places and people. ‘People are struggling to breathe, and more so in some places than others’, as Kim Fortun says (Fortun, 2014, p. 326; Nunn, 2018; Roberts et al., 2008).

But things are perhaps even more complicated as the interrelatedness of life and death does not only affect differentially and disproportionately certain groups of humans and nonhumans but increasingly most human and nonhuman bodies as many substances have planetary reach and simultaneously sustain and cause bodily harm. Security and risk, invigoration and destruction coincide in human bodies, our neighbourhoods, our ecologies, our worlds. The pervasiveness of anthropochemicals implicates social groups and places which otherwise seem less vulnerable than those which are disproportionately affected by toxicity and pollution.

Despite this inextricable link between chemicals, ecologies and societies, there is a broad underlying assumption that anthropochemicals can be eventually detached and the social and political body can be “purified”. There is a widespread belief that our societies can be separated from the toxic chemicals that they produce. There is often an implicit assumption to this belief: that the proliferation of anthropochemicals is primarily the outcome of economic interests. Chemicals have been simultaneously the source and the product that drives multiple operations in human production systems. The consequence of this approach is that the current capitalist mode of production is elevated to a singular historical subject of agency that has the capacity to adopt (or ideally reject) the production and use of harmful substances. Chemicals are implicitly conceived here as both constraints of human action and simultaneously as action enabling tools that can be discarded if the system would decide to do so. These two poles represent two widespread popular understandings of science and technology that have been extensively discussed within Science and Technology Studies: technoscientific determinism on the one hand and the political determination of technology on the other (for a discussion of both see Winner, 1980; Wyatt, 2008). Common to both is that knowledges, materials and technologies – including anthropogenic chemicals that I discuss in this paper – are made to be the mere object of human will. Within this unreflectively humanist and anthropocentric view of our worlds, materials and technologies represent the ontologisation of will.

Anthropochemicals are so deeply and inextricably linked to human societies that the idea that they can be simply disengaged from human bodies, human technologies and ecologies by some form of global political will is evidently impossible. But even if in a thought experiment human societies would be able to arrive at a global decision to eradicate harmful chemicals, they would be faced with the insurmountable problem of the mundane spread of chemicals: technoscientific objects, the bio- and geosphere, and human everyday life are linked to each other in ways that are ungraspable and, indeed, ungovernable by human politics whether there is political will or not. Anthropogenic molecules are produced in so many different multifaceted processes and contexts that rather than being explainable through an economistic approach to the current mode of production, they can perhaps help us explain the function and system of production itself. When it comes to chemicals, the capitalist mode of production is not the explanans but the explanandum.

Anthropochemicals implicate us because they cut across and reconfigure power divides, social asymmetries, political injustices, ecological imbalances, and material conflicts in multiple ways (Papadopoulos et al., 2021). As much as chemicals are a core component of the supply chains and production networks of contemporary Global North societies, they are also intrinsic to many material, cultural, psychosocial and biosocial ways of being that cannot be disentangled from one another. Anthropochemicals implicate humans because they enable them in so many ways that they are impossible to be dissociated from their damaging effects. Almost every anthropochemical is simultaneously enabling and damaging. This entanglement is relentless and there is no possibility of erasing the presence of chemicals in our societies. So, what is the escape route from this unavoidable grip of human societies by chemicals? Anthropochemicals implicate us and we need to remake them differently in order to escape them. We escape with them rather than from them. In a sense the antidote to chemicals are chemicals. A utopian scenario would be a radical programme for replacing all current chemicals with fully benign substances and this can be a programme that starts from the individual level through the community level all the way to societal governance. It has become impossible to maintain life without anthropochemicals. In these conditions the transition to anthropogenic benign chemicals has become an imperative (Anastas & Zimmerman, 2018).

In what follows I want to discuss the condition of being implicated by anthropochemicals and the imperative to replace harmful substances with sustainable and benign chemicals through the problem of growth and scale. The scale of production and use of harmful chemicals is intimately linked to the continuous growth of human societies. A no growth alternative appears to be the only reasonable solution. However, the size of any programme that would materialise the transition to sustainable and benign chemicals requires a transformation of such a scale that complicates the polarity between growth and no growth. What is an appropriate scale of change that can enable the social transformation towards sustainable chemicals? How can we imagine our present otherwise and our worlds composed differently (Savransky, 2021b) when a clean up of our damaged ecologies and an ‘after’ to our polluted environments is impossible? In the following section I will discuss how the problematic of scale complicates our understanding of growth and questions the binary growth/no growth. In the third section I will move to explore how scale is embedded in an ecological milieu and how the experience of ecology can drive the reparation of damaged environments. Finally, in the concluding section I will introduce some alterative conceptualisations of scale that may contribute to a discussion of the transition to sustainable and benign chemicals.

## The problematic of scale and the double limits of growth

In practices that involve chemicals everything is about scale. Scale embodies the powers and the pitfalls of chemistry and although it is probably true that there is chemistry without scale, it also true that there cannot be modern chemistry without scale (Bensaude-Vincent, 2013; Bensaude-Vincent & Simon, 2008; Klein, 2005; Lefèvre & Klein, 2007). The difference is minimal and yet has considerable implications. Modern chemistry is predicated on the *imperative of transcendence*: Transcending the obstacles in the manufacturing of a molecule in order to make it widely accessible. The drive for transcendence makes modern chemistry a typical technoscience. In the technosciences the main concern is to overcome the concrete barriers that emerge in the making of novel substances, materials or technologies and then step by step, challenge by challenge, obstacle by obstacle to scale them up through the manufacturing process so that they circulate through economy, society, and everyday life. In the technosciences, and of course in modern chemistry as one of them, the loops between research and potential applications, between knowing and making, between the multiple human and nonhuman actors that participate in the creation of science, and between research experimentation, technological design, the production process and everyday life are short and polyvalent (Bensaude-Vincent, 2013; Bensaude-Vincent & Loeve, 2018; Haraway, 1997; Ihde & Selinger, 2003; Ravetz, 2006).

As much as modern chemistry is driven by the imperative of transcendence it is also an empirical-practical science. Transcendence is resolved and achieved on each singular specific step along the way from making a novel substance to its application; and if it is not achieved within each step then transcendence collapses: the molecule is no longer produced and vanishes into the scientific archive, research papers or engineering logbooks. The empiricism of modern chemistry lies with the fact that it solves the challenges and obstacles it encounters always internally. It does not follow an external plan or grand theory but a myriad of mundane attempts to negotiate and settle uncertainties, complications, and difficulties that emerge at every specific step in the making and production of a molecule. Paradoxically the imperative of transcendence is always dealt with immanently. Modern chemistry is a radically empiricist science that constantly strives to transcend the scale of what it makes.

Between 2019 and 2022 I conducted as a Leverhulme Fellow an ethnographic fieldwork study on the emergence on green and sustainable chemistry. When I mentioned the empirical nature of modern chemistry to a leading chemist on ‘catalyst discovery’, he said: ‘if you read an academic paper in chemistry it makes it sound as though it’s all worked out that we had some hypothesis, we had some target molecule we were doing and we went in there and did it and of course it virtually never happens like that, . . . we may have a process in mind and we design a catalyst we think is going to work and you know, part of that is calculation and part of it of what people have done before but you know, more than half of it is just our intuition for it kind of this feels like it might be the right thing to do and most of the time they don’t work and so what do we then? Well certainly what we do in my group is say “Okay, well it’s not worked for this process but why don’t we try it for this or why don’t we try it for that?”’ (Field interview B331). And as much as this mundane empiricism is what constitutes research in chemistry, the drive and challenge is to scale up the production of molecules. The same scientist told me: ‘as scientists we can keep discovering new, interesting things and, you know, new specific transformations, new solvents. Of course, there are scientific challenges in this, you know, I’ve mentioned one, to develop catalysts that are tolerant of the sort of feed stocks we’d want from biomass. But I think those scientific challenges are less important than the challenge of scaling some of this up, of getting genuine investment in this and having real commercial technology that we can use as an exemplar for the success of this area, the sort of thing that you can show the Minister around to show that this is really delivering for the UK economy and delivering for sustainability and is based on fundamental science that was developed in the UK. I think if we can do that to champion the area then that’s more important than the scientific challenges that remain’ (Field interview B331).

‘Was the reaction atom economic?’ (Field interview C328); ‘Do you need to get a pure chemical from this process?’ (Field interview Y313); ‘How many kilograms of waste per kilogram of product does the reaction generate?’ (Field interview A334-5); ‘How much do we reduce energy consumption by using supercritical CO^2^?’ (Field interview N330); ‘What are these products in the material that we discovered through chromatography and that were not there before?’ (Field interview C328); ‘How can we minimise the variability of the feedstock?’ (Field interview Y371); ‘How can I reduce the steps of the reaction?’ (Field interview C350); ‘How are you going to control the heat of the reaction?’ (Field interview B332); ‘Do I know enough about the calorimetry and the kinetics of my new polymer forming reaction so that it can scaled up later?’ (Field interview N367); ‘How can we reduce the size of the reactor to increase safety and efficiency?’ (Field interview N402); ‘Is there a way that you can redesign a reaction to use an alternative greener solvent?’ (Field interview L319) – these are just a few questions extracted from conversations during the lab fieldwork that I conducted as part of the research study I mentioned earlier that exemplify the inherent issues that emerge in each specific step of the invention process of a molecule all the way to its wider manufacturing and point towards the empiricism of modern chemistry. With Deleuze and Guattari (1994, p. 47) we could say that this is a radical empiricism: ‘When immanence is no longer immanent to something other than itself it is possible to speak of a plane of immanence. Such a plane is, perhaps, a radical empiricism.’ But this radical empiricism of modern chemistry is always paired with the drive to scale up. Scalability is transcendence and transcendence means moving beyond the inherent limits of a specific reaction on a specific scale by staying within the given constraints of this specific scale and overcoming the obstacles that emerge within it. In modern chemistry the immanence of research materialises transcendence and manifests scale.

It is this riddling and improbable coexistence of transcendence and immanence that necessitates to reconsider what is scale in modern chemistry. Scale here does not only mean measure (of volume, mass, intensity, quality or value) but also refers to a specific plane on which research is undertaken and issues are resolved. This plane of immanence of every specific scale in the life of a molecule constitutes the *ecological milieu* that sustains a molecule of a certain quantity and the actual process of its making. On each and every level of scale in the life of a molecule a different ecological and material milieu either secures its further existence or is unable to create the right order and terminates the potential of a molecule to become widely accessible across society leaving only a traceable existence in chemistry research papers and perhaps the process design briefs of engineers.

Consider the case of the urgently needed transition to safe alternatives of hazardous solvents – the non-active substances in countless commercial and industrial solutions – which introduces a series of complex social and material issues at each one of the different phases of their development (Jessop, 2016; Poliakoff & Licence, 2015). For example, the alternative solvent dihydrolevoglucosenone which was invented at the Green Chemistry Centre of Excellence, University of York (Clarke et al., 2018) is a novel compound derived from biomass and food waste that could be used as replacement for NMP, a widely used organic compound which is a known substance of very high concern because of its environmental and health safety effects. The conditions that the novel alternative solvent assembles around itself when created at labscale are very different from the conditions of the compound at demonstrator or production scale. As the compound changes scale a very different set of issues and actors sustain its existence. As dihydrolevoglucosenone moves to production scale issues of standardisation, toxicity metrics, public regulation, innovations in process engineering, industrial entrepreneurship, economic and financial viability and, perhaps most importantly, the sustainable access to biomass become crucial dimensions of its existence. As waste and biomass become a valuable resource in their own right (Dugmore et al., 2017; Poliakoff et al., 2002; Sheldon, 2007; Tuck et al., 2012) a series of questions about a holistic approach to value emerge (Gupta et al., 2018) that encompass the sourcing of feedstocks, energy efficiency, and the afterlife of the solvents (Caillol, 2013; Clark et al., 2016; Kümmerer, 2007).

Every step in changing the scalability of a chemical, every step in extending its scale does not only involve the modification of measure but also the immanent alteration of its ecological milieu. Thus, even if scale appears to be an extension of a linear progressive system of classification it necessitates something much more diverse and complex in order to exist: it necessitates a whole ecology of practices (Stengers, 2021), processes and objects that allow a molecule to exist within the ecological milieu of a specific scale. Such ecologies are complex arrangements that involve a multiplicity of human actors, engineers, chemists, local communities, governing bodies and regulatory authorities, industry and, of course, a multiplicity of nonhuman actors, objects, landscapes, and other species.

Scale is crucial for chemical practice because for the ‘same’ compound in different measures there is a very different ecology that is involved. Depending on how much this ecological question of scale becomes considered or is erased, the transition to sustainable and benign compounds that incorporate such ecological concerns becomes a more or less urgent question. Scale is, as I mentioned earlier, crucial for the making of modern and contemporary chemistry, but the problematisation of scale is even more crucial for creating alternative green and sustainable compounds. Throughout my fieldwork in green chemistry labs and within the extensive series of interviews with preeminent green and sustainable chemists^1^ scale appears as one of the most salient and equally controversial dimensions for green and sustainable chemical practice. What kind of scale do we need? How much of it and how? But probably one thing that seemed to represent most if not all the positions is that there cannot be change in chemistry without scale. So, scale appears to be both the problem and the solution.

So, how much scale do we need to change things? What is the scale of chemicals that is needed if chemicals always implicate us? The problem with scale is that it is an ambivalent concept, we need scale to encounter the depth and width of ecological destruction and simultaneously we know that scale is the engine of productionism and productionism drives growth which is a major cause of ecological destruction. In a shortcut, scale is linked to destruction. And yet, there is something plausible about scale: without scale there is very limited scope for meaningful ecological change. Scale is an ordinary concept as it speaks to a sense of planetary belonging that corresponds to the need of a significant politics of scale to reduce carbon emissions, reverse geo- and biodiversity loss, eliminate pollution and toxicity, and instigate transformative societal programmes.

The urgency of a transition to green and sustainable chemicals exemplifies this Janus-faced meaning of scale: scale is tightly connected to environmental destruction but it is also an indispensable component of environmental action for avoiding catastrophic futures. Within social thought approaches to scale tend to conceive it as a theoretical problem, in fact as something that might not even exist as such. Strathern (2000), Bruun Jensen (2015) and others, for example, make the case that rather than nesting scales we have fractal environments whose complexity is scale invariant as they incorporate many different qualities that traditionally belong to different scales. Tsing (2012, 2015) goes a step further and elevates scalability to a device that performs domination and ultimately social and ecological destruction. Thinking scale with chemicals complicates these approaches: toxic, hazardous and primarily petrobased anthropochemicals are produced and used at such scale that we need a tremendous economy of scale of alternative chemicals in order to replace them. Scale = ecological degradation. Simultaneously: no scale, no ecological transition. As much as scale is historically and geopolitically embedded in a certain form of social organisation, it now exceeds this determination to manifest itself ontologically in the degradation of our ecologies and, simultaneously, in any vision of ecological transition.

Rather than a single issue to be decided for or against, this ontological pervasiveness and inherent ambivalence of scale makes it a problematic (Leistert & Schrickel, 2020; Savransky, 2020): Scale is the organising principle of ecological degradation and simultaneously it is a necessary component of many reparation and remediation attempts. Without some form of scale there is no possibility to confront the extent to which anthropogenic chemicals pervade everything. The fact that human societies are so deeply implicated by chemicals renders this ambivalence of scale all the more present and, indeed, vital. It is this link to the vital threats but also vital possibilities that scale incorporates that makes it so valuable for political strategists of every kind and taste who try to harness current widespread environmental unease and respond to today’s ecological urgency: First, those who use scale as a proxy to revolutionary system-wide change in order to overcome capitalist productionism and only alleviate ecological damage afterwards; second, those ultra-neoliberals who use scale to instigate new modes of value creation that largely ignore or are opportunistic towards ecological damage and environmental change; third, those statists who need scale to preserve the elitist make-up of liberal Global North societies through the implementation of a technofix-driven green deal; and fourth, those autocrats and regressive nationalists who use scale to consolidate and expand their power by negating the environmental effects of scale altogether. The ambivalence of scale makes it easily compatible with so many divergent approaches: the revolutionaries, the neoliberals, the liberals, the autocrats (Ghelfi & Papadopoulos, 2022b). And, of course, this ambivalence pertains also to a fifth group: those who refuse to engage with scale altogether because of the danger of co-option. In this view current societies cannot but appropriate scale to reinforce existing power asymmetries and thus any thinking of scale is impossible even if, paradoxically, we think with the notion of scale to describe the grave social and environmental problems that we encounter. In all these five very different positions, the problematic of scale is that you need it but when you have it, it undermines transformational socio-ecological change.

We are implicated by scale both in its destructive pervasiveness and its promissory capacities. This duplicitous character of scale emanates from its deep attachment to economic growth as the single most powerful driver of environmental destruction. But the problematic of scale complicates our relation to growth when it comes to ecological questions today and, in particular, to the pervasiveness of harmful chemicals which is the focus of this article. The scale of toxic chemicals and the scale of alternative benign chemicals that are needed to reduce further ecological degradation sit uneasily with both, the celebration of growth and its outright dismissal. Being implicated by scale means that chemicals and materials production cannot be just stopped, they need to be replaced with other chemicals even if slow growth or degrowth were possible. Growth together with scale are at the heart of the problem of the proliferation of harmful anthropochemicals and simultaneously they need to play a key role in their replacement.

From a materialist perspective on anthropochemicals, limitless growth and no growth, fast and slow, large scale and small scale become impossible to be decided in practical terms, they are ‘infernal alternatives’ (Pignarre & Stengers, 2011). The category of growth is too universal to be encountered. Its absence mirrors the universality of its presence. If its presence is associated with ecological degradation and loss of nonhuman life, its absence is imposed on those who already feel its destructive consequences: disadvantaged and marginalised social groups, the Global South and large ecosystems and other species. While growth is unevenly and unequally distributed across different regions and spaces of the planet, imposed economic no growth has similar devastating social and ecological consequences to the places that experience it. Steady growth has been always a historical exception reserved for small parts of the Global North and an often vacant promise for so many other places, while the absence of growth or imposed no growth has been the norm for large parts of the Global South and occasionally also in certain parts of the Global North.

The limits of growth extend in two directions: there is always too much growth and there is not enough to prevent conflict and decay. We are in a moment in which growth brings environmental and social destruction and when it slows down it brings pain, deep social conflicts (that on many occasions release fascist-oriented political movements) and ecological destruction (Ghelfi & Papadopoulos, 2022b). From the perspective of chemicals, the absence of growth does not lead to the absence of harmful substances but to the continuation of their proliferation: two literally ‘infernal’ alternatives. When it comes to chemicals the absence of growth does not mean no growth and reduction of ecological impact, it means a regress to a form of chemical practice and production that continues to neglect its ecological obligations. Scale as well as no scale, growth as well as no growth constitute a negation of the ecological embeddedness of anthropochemicals and their production, use and disposal.

## The bifurcation of ecological experience and ecological reparation

I mentioned earlier in the article that scale is not just about measure but about the incorporation of the ecological context into the life of a molecule. When scale/no scale become the only two existing options mirroring the infernal alternatives of growth/no growth, what suffers is the ecological milieu that houses scale. What does the loss of ecology specifically mean in the binary opposition between scale/no scale and growth/no growth? The loss of ecology involves the loss of the continuity between the many distinct registers that constitute what the ecological is: (1) the epistemic register of ecology as a scientific discipline;^2^ (2) the ontic register, referring to the relations and becomings among beings doing life together; (3) the lived register of the everyday experience of rootedness and belonging in our surroundings and the embodied understanding of our worldly connections with different beings and spaces; (4) the economic register, which approaches ecology through the lens of value and conceives ecosystems as distinct worldly productive entities; (5) the political register, which involves a multiplicity of social movements and a long tradition of environmental activism and ecological mobilisations; (6) and, finally, the cultural register, from ecological urbanism to the alternative cultures of rural resurgence, from environmental fine art photography to ecopoetry, from ecology as a mode of thought to ecology as a method of enquiry.

All these registers together constitute ecology as experience, a ‘general ecology’ (Hoerl & Burton, 2017), that is generative (Figueroa Sarriera & Gray, 2016) of experiences and social relations that put the ecological at the heart of worldly existence. The general experience of ecology is not to harmonise the relations between all these different registers that are often in conflict with each other. Rather, in line with an ecosophical approach in Guattari’s (1995, p. 91) terms, this is an attempt for a ‘generalised ecology’ that tries to articulate and re-articulate ‘scientific, political, environmental and mental ecologies’ (1995, p. 134; see also Hoerl, 2013). Guattari’s ecosophical project of a generalised ecology echoes Bateson’s (1972) holistic and relational approach to how human mind, technology and the environment are inseparably entwined. There is no dominant subjectivity here, there is no primacy of the human, the socio-technical or the environmental. They all exist on the same plane and constitute the continuous experience of ecology. Such an a-subjective approach breaks away from humanist understandings of the ecological that see humans realising themselves if only they abandon technology, enlarge their identification with broader ecologies, and reinstall a harmony with the natural world – such as in deep ecology accounts in Fox (1990), Mathews (1991) and Naess (1973) (for a discussion see Bogue, 2009; Braidotti, 2006).

Guattari’s ecosophical approach is about how the uneasy and conflictual dynamics of the relations between humans, societies, technologies and ecologies create an everyday ‘continuous experience’ (Stephenson & Papadopoulos, 2006) of ecology (see also Brown & Stenner, 2009; Brown et al., 2011; Schraube & Højholt, 2016). When the continuous experience of ecology is split, ecology is dominated by one singular register. A holistic and continuous experience of ecology is a quest rather than a given. It almost feels an impossibility in today’s social and environmental conditions in which ecology bifurcates to different registers. Similar to Whitehead’s (1964) ‘bifurcation of nature’, which laments how modern thought splits the experience of the world into primary qualities – objective and scientific – and secondary qualities – psychological and cultural – (Debaise, 2017; Goffey, 2008; Haas, 2018; Halewood, 2012), the bifurcation of ecology splits the experience, practice and process of ecology into a series of dualisms between distinct registers, each one of them operating independently and often against each other.

The chasm between these different ecological registers shapes the perception of the world excluding or even actively erasing other ecological registers and making certain registers appear as facts. As Whitehead argued: ‘The world is not merely physical, nor is it merely mental. Nor is it merely one with many subordinate phases. Nor is it merely a complete fact, in its essence static with the illusion of change. Wherever a vicious dualism appears, it is by reason of mistaking an abstraction for a final concrete fact’ (A. N. Whitehead, 1967, p. 190). The lived ecological register during a countryside hike might be experienced as opposite to the political domain for environmental justice, the ontic register of cohabitation of a certain terrain might be experienced as irrelevant to the economic productivity of its ecosystems, and so on. Accepting the current scale of environmental destruction is only possible because of the bifurcation of ecology as different ecologies diverge from each other. The restoration of a general and generative experience of ecology remains a quest that is gradually becoming the drive of many contemporary social movements (Ghelfi & Papadopoulos, 2022a, 2022b).

Anthropochemicals have been a matter of concern for many decades now but it is only recently that they have also become the site of a promise, even if only a minor one. What if the chemicals humans create and use can be made differently? What if we look for substances that are less harmful outside of chemical science? What if chemistry itself can transform to become aware and incorporate its ecological milieu in its own making? Against the imperative of transcendence and the loss of the continuous experience of ecology there is a multitude of attempts to bypass the double impasse of the scale/no scale binary and to explore how chemistry can gravitate around a general ecology: from green and sustainable chemistry to the amateur science of alternative chemical practices, from traditional knowledge systems to community technoscience, chemicals increasingly become a site for experimentation with their ecological implications.

Chemicals become obliged by ecology. Puig de la Bellacasa (2015; 2017, pp. 150ff.) highlights that obligation is not only about a sense of moral responsibility but it primarily involves a practical-material dimension and the urgency for action: being obliged by ecology means that one has no choice but to care for and repair the ecologies one is part of. A wide range of community projects, social movements and environmental justice campaigns move beyond their single focus on protest politics to engage with transforming the materialities in which they find themselves. This multitude of ‘more-than-social movements’ (Ghelfi & Papadopoulos, 2022a; Papadopoulos, 2018) practise ecological reparation both as repair of damaged ecologies and claiming reparations for the ecological wrongdoings that these human communities and the nonhuman worlds in which they exist have suffered (Papadopoulos et al., 2022). They move beyond more visible institutionalised forms of reconciliation and state reparation – such as large scale compensation policies, formal exchanges between representatives of conflict groups, widespread provision of psychosocial assistance, ceremonial commemorations – to instigate forms of reparation that attempt to restore the cohabitation of the human and the nonhuman world (Cadieux et al., 2019; Cairns, 2003; Caney, 2006; Hale et al., 2014). Inspired by reparative justice (Macleod, 2019; Mora-Gámez, 2016; Perez Murcia, 2013; Walker, 2010; White, 2016), ecological reparation redirects reparations to the repair of interspecies and human–nonhuman relations damaged by racism, extractivism and environmental injustice. Repair is a vital process within ecological reparation (Darwish, 2013; Denis, 2019; Jackson, 2014; Tacchetti et al., 2022): repair as the unfolding of relations, movements and interconnections among diverse humans and nonhumans in the patching up and reclaiming of damaged socio-environmental ecological niches.

## Conclusion: Scaling out

So, this is my question: growth aside, what do we do with the planetary and social boundaries to scale that we are currently facing? There are many different conceptualisations of planetary boundaries (e.g. Diamond et al., 2015; Rockström et al., 2009) that point towards the necessity for a different practice of scale. Neither as an answer to growth nor as an alternative to it but because the presence of growth has made any meaningful engagement with social and planetary boundaries impossible. Rather than embracing any of these impossible alternatives that I mentioned earlier, many social movements attempt to create conditions for establishing their own boundaries and installing their own scales (Kallis, 2019), and to become attentive to practices and stories beyond the dominance of growth (Calvário et al., 2022; Savransky, 2021a). The process of constructing these adequate scales is a process of *commoning* the boundaries of socio-ecological life: instead of adopting some external imposed boundaries, many communities negotiate and, most importantly, practise other scales that ascertain ecological boundaries to communal life. This is eco-commoning – groups of humans and nonhumans make and uphold communally maintained spaces and ecologies in order to reclaim and repair damaged ecologies (Bresnihan, 2013; Castellano, 2017; Ghelfi, 2015; Linebaugh, 2008, 2010; Papadopoulos & Puig de la Bellacasa, 2022; Papadopoulos et al., 2022; Reid & Taylor, 2010; Wall, 2014).

There are many examples of such initiatives that often involve experimenting with alternative materials, substances and anthropochemicals. Consider for example urban experimental eco-living (Pickerill, 2020), the creation of community managed localised infrastructural provision (Hodson et al., 2018), alternative energy supply (Angel, 2017), applied degrowth campaigns (D’Alisa, 2015; Demaria et al., 2013; M. Whitehead, 2013), indigenous forms of eco-social life (Mander & Tauli-Corpuz, 2006; Whyte, 2018), post-developmental politics (Escobar, 2015), urban farms (Salvatore Engel-Di Mauro, 2022), environmental justice campaigns (Agyeman et al., 2016; Bullard & Wright, 2009; Dillon, 2014), ecological activism (Gatt, 2017), maker movements (Ottinger & Cohen, 2011), decolonial ecologies (Ferdinand, 2019), post-capitalist economies (Gibson-Graham, 2006), transition towns (Hopkins, 2011), food sovereignty movements (Shattuck et al., 2017), permaculture gardens (Mars et al., 2016), commons transition (P2P Foundation, 2015), climate urbanism (Bulkeley, 2015), environmental citizenship (Dobson & Bell, 2006), social and solidarity economies (Utting, 2015), bioregeneration (Darwish, 2013), the peasant confederation La Via Campesina and agroecology (Rosset, 2017). What is common to all these very diverse examples is that they establish their own planetary and ecological boundaries as they engage in the ecological reparation of the spaces that they inhabit and maintain.

Ecological reparation is inspired by the practices of these and many other social movements that operate with, within and often against instituted technoscience by engaging with the double bind of scale: for many of these movements scale is not about replicating and multiplying the same type of action in order to create change. Rather it is about engaging with the challenge of ecological reparation and developing alternative ontological conditions of existence, *alterontologies* (Papadopoulos, 2018), on the terrain on which each one of these movements and communities live. Rather than copying and repeating the same practice to scale it up, alterontologies proliferate in intensive ways on the everyday life of communities. Experimentalism is not about replication – something already discussed extensively in Science and Technology Studies (Collins, 1985; Hacking, 1983; Knorr-Cetina & Mulkay, 1983). For replication to happen and to create scale a process of delocalisation and the erasure of the ecological milieu is necessary – something that I have discussed earlier in this article in the case of modern chemistry as the imperative of transcendence. Operationalise, purify and transcend many of the actual conditions that made the experiment possible. Scale becomes a model that dominates many locales.

Scaling *out* promotes an alternative approach to scale as replication and domination: different alterontological experiments emerge in different communities and many of these despite their significant differences align with each other to practise ecological reparation and create alternatives on the ground. Consider for example the repair and remediation of chemically polluted ecosystems, in particular through bioremediation technologies. Bioremediation is typically conceived as ‘a *natural process*, which relies on bacteria, fungi (mycoremediation), and plants (phytoremediation) to degrade, break down, transform, and/or essentially remove contaminants, ensuring the conservation of the ecosystem biophysical properties’ (Masciandaro et al., 2013, p. 399). Other similar definitions emphasise ‘*harnessing* the degradative potential of biological systems’ (Cummings, 2010, p. v). Bioremediation relies on the processes of a nonhuman world: ‘Without the activity of microorganisms, the earth would literally be buried in wastes, and the nutrients necessary for life would be locked up in detritus’ (Bonete et al., 2015, p. 24). Therefore the use of bioremediation techniques is often seen as ‘working with nature’ – a trope shared by grassroots, scientific and industrial bioremediation projects (Bharagava, 2017).

Yet bioremediation technologies are not ‘natural’ or merely ‘biological’ but naturecultural and socio-technical. They integrate sophisticated lab-based research and applied environmental biotechnology to accelerate the degradation of polluting contaminants. While industrial bioremediation is often based on a clean up and leave approach neglecting follow up and community maintenance as well as the wider effects on local environments and other species, community supported bioremediation projects are not only about cleaning up contaminants but a way of reintegrating anthropogenic chemical practice – including labouring, affective and ethical aspects – in natural cycles by collaborating across different actors and species (Puig de la Bellacasa, 2021). Reparative alterontological bioremediation projects are always situated and context specific and involve interspecies relations of care while simultaneously relying on the circulation of translocal bioremediation knowledge and practice (Darwish, 2013; Hartigan, 2015; Paxson & Helmreich, 2013; Puig de la Bellacasa, 2010, 2012, 2017).

The ecological reparation of such experiments is not to return back to a state free of anthropochemicals. There is no restoration of a prior state of being; neither is it possible to compensate for the damage that anthropochemicals cause on humans, nonhuman others and the environment. Ecological reparation here means creating alterontologies driven by the continuous experience of general ecology. Ecological reparation is experimenting with chemical substances that install a different lived experience of ecological cohabitation. Are these alterontological practices enough to create sweeping societal change? Perhaps at some point, but possibly not. They are enough though to defend and maintain the life of communities facing social-ecological conflict and destruction. Alterontologies are not the same as prefigurative politics that aim to realise some parts of the desired future in the present (Chatterton & Pickerill, 2010; Graziano, 2016; Pickerill & Chatterton, 2006; van de Sande, 2013). Alterontologies do not primarily point towards some sort of other global politics of transformation to come. There is no ‘post’ in alterontological politics. Their intensive and experimental material engagements in the present is all there is.

Scaling out involves considering the ecological in many different locales, starting from the green and sustainable research in chemistry science labs and in chemical engineering workshops and moving to a multitude of transformative engagements with anthropogenic chemicals in a plethora of spaces outside of the instituted science of chemistry itself. Chemical practice becomes dispersed in society: the creation of ‘translocal infrastructures’ (Ghelfi & Papadopoulos, 2022a) and the ‘distributed invention power’ (Papadopoulos, 2018, p. 182) of amateur scientists, indigenous knowledge practitioners, clandestine chemists, DIY biochemists, researchers in green and sustainable chemistry, remediation ecologists, biodegradable designs, underground labs, interspecies collaborations; entheogens and healing compounds, ethnobotanical knowledges and kitchen chemistries, baking bread, making beer, mattering compost; making amateur-led pollution sensing devices, monitoring chemical toxicity, creating vocabularies, images and stories to capture life in contaminated worlds, doing independent chemical experimental lab work, incorporating green chemistry within citizen science – generative chemical practice as an integral part of technoscience that travels and connects the lab, the engineering bench and community projects. Amateur and professional chemical practitioners create alternative ontologies, alterontologies, on the molecular level. There is a dense traffic between the scaling-up practices of instituted technoscience such as green and sustainable chemistry on the one hand and the scaling-out practices of community-based generative chemical practice on the other hand. Rather than opposition and exclusion, instituted green and sustainable chemistry is one of the currents that are necessary for scaling out chemical practice horizontally as they challenge established research norms and customs within chemistry itself. The proliferation of distributed transformative practices through community specificity, material singularity and practical concreteness is what creates change: many alterontological practices. Change, even if minor, emerges from creating alternative ways of existence obliged by the experience of ecology that give birth to novel ecologies, and possibly also to worlds that do not yet exist (Lundy, 2019). The political significance of alterontological chemical practices emerges from the fact that they engage technoscience and other traditional forms of knowledge to secure communal life in midst of socio-ecological conflict.

## Notes

1. Within the discipline of chemistry itself we have for example green and sustainable chemistry that emerged in the 1990s and aimed to create waste-free, non-toxic, low impact chemical compounds (Anastas & Warner, 1998; Clark et al., 2014; Sheldon, 2016). Rather than the common attempt to limit and regulate the handling and effects of end-of-pipe chemicals of which only very few are already environmentally friendly, green and sustainable research focuses on a ‘benign by design’ approach that seeks to limit or even eliminate negative environmental consequences already in the process of synthesising molecules (Sanderson & Anastas, 2011). By the end of the 1990s a series of prominent initiatives had helped establish this line of research, such as the US Environmental Protection Agency’s Green Chemistry Program, the Green Chemistry Network and the associated journal of the Royal Society of Chemistry in the UK (Anastas et al., 2016; Iles, 2013; Linthorst, 2010; Woodhouse & Breyman, 2005). It is only in the end of the 2000s that the field started to have a wider impact.

2. Although it is important to highlight here that ecological science is in itself very diverse and is intimately embedded in wider ambivalent social, environmental and historical settings, with its historical beginnings linked to colonial expansion, travel writing, and assuming the world from the explorer’s perspective (Ferdinand, 2019; Gómez-Barris, 2017; Pratt, 1992). The ecological sciences were invented, at least in North America, as part of a larger socio-epistemic movement that facilitated colonisation and extraction of native lands (see for example Kingsland, 2005).

## References

[bibr1-00380261221084780] Agyeman J. Schlosberg D. Craven L. Matthews C. (2016). Trends and directions in environmental justice: From inequity to everyday life, community, and just sustainabilities. Annual Review of Environment and Resources, 41(1), 321–340. doi:10.1146/annurev-environ-110615-090052

[bibr2-00380261221084780] Anastas P. T. Han B. Leitner W. Poliakoff M. (2016). ‘Happy silver anniversary’: Green Chemistry at 25. Green Chemistry, 18(1), 12–13. doi:10.1039/c5gc90067k

[bibr3-00380261221084780] Anastas P. T. Warner J. C. (1998). Green chemistry: Theory and practice. Oxford University Press.

[bibr4-00380261221084780] Anastas P. T. Zimmerman J. B. (2018). The United Nations sustainability goals: How can sustainable chemistry contribute? Current Opinion in Green and Sustainable Chemistry, 13, 150–153. doi:10.1016/j.cogsc.2018.04.017

[bibr5-00380261221084780] Angel J. (2017). Towards an energy politics in-against-and-beyond the state: Berlin’s struggle for energy democracy. Antipode, 49(3), 557–576. doi:10.1111/anti.12289

[bibr6-00380261221084780] Barry A. (2005). Pharmaceutical matters: The invention of informed materials. Theory, Culture & Society, 22(1), 51–69. doi:10.1177/0263276405048433

[bibr7-00380261221084780] Bateson G. (1972). Steps to an ecology of mind. Ballantine.

[bibr8-00380261221084780] Bensaude-Vincent B. (2013). Chemistry as a technoscience? In Llored J. (Ed.), The philosophy of chemistry: Practices, methodologies, and concepts (pp. 330–341). Cambridge Scholars Publishing.

[bibr9-00380261221084780] Bensaude-Vincent B. Loeve S. (2018). Toward a philosophy of technosciences. In Loeve S. Guchet X. Bensaude-Vincent B. (Eds.), French philosophy of technology: Classical readings and contemporary approaches (pp. 169–186). Springer.

[bibr10-00380261221084780] Bensaude-Vincent B. Simon J. (2008). Chemistry: The impure science. Imperial College Press.

[bibr11-00380261221084780] Bensaude-Vincent B. Stengers I. (1996). A history of chemistry. Harvard University Press.

[bibr12-00380261221084780] Bharagava R. N. (Ed.). (2017). Environmental pollutants and their bioremediation approaches. CRC Press.

[bibr13-00380261221084780] Bogue R. (2009). A thousand ecologies. In Herzogenrath B. (Ed.), Deleuze/Guattari & ecology (pp. 42–56). Palgrave Macmillan.

[bibr14-00380261221084780] Bonete M. J. Bautista V. Esclapez J. García-Bonete M. J. Pire C. Camacho M. Torregrosa-Crespo J. Martínez-Espinosa R. M. (2015). New uses of haloarchaeal species in bioremediation processes. In Shiomi N. (Ed.), Advances in bioremediation of wastewater and polluted soil (pp. 23–49). Intechopen.

[bibr15-00380261221084780] Boudia S. Creager A. N. H. Frickel S. Henry E. Jas N. Reinhardt C. Roberts J. A. (2018). Residues: Rethinking chemical environments. Engaging Science, Technology, and Society, 4, 165–178. doi:10.17351/ests2018.245

[bibr16-00380261221084780] Braidotti R. (2006). Transpositions: On nomadic ethics. Polity Press.

[bibr17-00380261221084780] Bresnihan P. (2013). John Clare and the manifold commons. Environmental Humanities, 3, 71–91.

[bibr18-00380261221084780] Brown S. D. Cromby J. Harper D. J. Johnson K. Reavey P. (2011). Researching ‘experience’: Embodiment, methodology, process. Theory & Psychology, 21(4), 493–515. doi:10.1177/0959354310377543

[bibr19-00380261221084780] Brown S. D. Stenner P. (2009). Psychology without foundations: History, philosophy and psychosocial theory. Sage.

[bibr20-00380261221084780] Bruun Jensen C. (2015, May 28–30). Mekong scales: Domains, test-sites, and the micro-uncommons. Paper presented at the Sawyer seminar workshop ‘Uncommons’, University of California, Davis.

[bibr21-00380261221084780] Bulkeley H. (2015). An urban politics of climate change. Experimentation and the governing of socio-technical transitions. Routledge.

[bibr22-00380261221084780] Bullard R. D. (1994). Dumping in Dixie: Race, class, and environmental quality. Westview Press.

[bibr23-00380261221084780] Bullard R. D. Wright B. P. D. (2009). Race, place, and environmental justice after Hurricane Katrina: Struggles to reclaim, rebuild, and revitalize New Orleans and the Gulf Coast. Westview Press.

[bibr24-00380261221084780] Cadieux K. V. Carpenter S. Liebman A. Blumberg R. Upadhyay B. (2019). Reparation ecologies: Regimes of repair in populist agroecology. Annals of the American Association of Geographers, 109(2), 644–660. doi:10.1080/24694452.2018.1527680

[bibr25-00380261221084780] Caillol S. (2013). Life cycle assessment and ecodesign: Innovation tools for a sustainable and industrial chemistry. In Llored J. (Ed.), The philosophy of chemistry: Practices, methodologies, and concepts (pp. 35–64). Cambridge Scholars Publishing.

[bibr26-00380261221084780] Cairns J. (2003). Reparations for environmental degradation and species extinction: A moral and ethical imperative for human society. Ethics in Science and Environmental Politics, 3, 25–32.

[bibr27-00380261221084780] Calvário R. Kaika M. Velegrakis G. (Eds.). (2022). The political ecology of austerity: Crisis, social movements, and the environment. Routledge.

[bibr28-00380261221084780] Caney S. (2006). Environmental degradation, reparations, and the moral significance of history. Journal of Social Philosophy, 37(7), 464–482.

[bibr29-00380261221084780] Carson R. (1962). Silent spring. Fawcett Crest.

[bibr30-00380261221084780] Castellano K. (2017). Moles, molehills, and common right in John Clare’s poetry. Studies in Romanticism, 56(2), 157–176.

[bibr31-00380261221084780] Chatterton P. Pickerill J. (2010). Everyday activism and transitions towards post-capitalist worlds. Transactions of the Institute of British Geographers, 35(4), 475–490. doi:10.1111/j.1475-5661.2010.00396.x

[bibr32-00380261221084780] Clark J. H. Farmer T. J. Herrero-Davila L. Sherwood J. (2016). Circular economy design considerations for research and process development in the chemical sciences. Green Chemistry, 18, 3914–3934.

[bibr33-00380261221084780] Clark J. H. Sheldon R. Raston C. Poliakoff M. Leitner W. (2014). 15 years of Green Chemistry. Green Chemistry, 16, 18–23.

[bibr34-00380261221084780] Clarke C. J. Tu W. C. Levers O. Brohl A. Hallett J. P. (2018). Green and sustainable solvents in chemical processes. Chemical Reviews, 118(2), 747–800. doi:10.1021/acs.chemrev.7b00571 29300087

[bibr35-00380261221084780] Collins H. M. (1985). Changing order: Replication and induction in scientific practice. Sage.

[bibr36-00380261221084780] Cummings S. P. (Ed.). (2010). Bioremediation: Methods and protocols. Humana Press Springer.

[bibr37-00380261221084780] D’Alisa G. De Maria F. Kallis G. (2015). Degrowth: A vocabulary for a new era. Routledge.

[bibr38-00380261221084780] Darwish L. (2013). Earth repair: A grassroots guide to healing toxic and damaged landscapes. New Society Publishers.

[bibr39-00380261221084780] Davies T. (2018). Toxic space and time: Slow violence, necropolitics, and petrochemical pollution. Annals of the American Association of Geographers, 108(6), 1537–1553. doi:10.1080/24694452.2018.1470924

[bibr40-00380261221084780] Debaise D. (2017). The modern invention of nature. In Hoerl E. Burton J. (Eds.), General ecology: The new ecological paradigm (pp. 151–168). Bloomsbury.

[bibr41-00380261221084780] Deleuze G. Guattari F. (1994). What is philosophy? Verso.

[bibr42-00380261221084780] Demaria F. Schneider F. Sekulova F. Martinez-Alier J. (2013). What is degrowth? From an activist slogan to a social movement. Environmental Values, 22, 191–215.

[bibr43-00380261221084780] Denis J. (2019). Why do maintenance and repair matter? In Blok A. Farias I. Roberts C. (Eds.), The Routledge companion to actor-network theory (pp. 283–294). Routledge.

[bibr44-00380261221084780] Diamond M. L. de Wit C. A. Molander S. Scheringer M. Backhaus T. Lohmann R. Arvidsson R. Bergman Å. Hauschild M. Holoubek I. Persson L. Suzuki N. Zetzsch C. (2015). Exploring the planetary boundary for chemical pollution. Environment International, 78, 8–15. doi:10.1016/j.envint.2015.02.001 25679962

[bibr45-00380261221084780] Dillon L. (2014). Race, waste, and space: Brownfield redevelopment and environmental justice at the Hunters Point shipyard. Antipode, 46(5), 1205–1221. doi:10.1111/anti.12009

[bibr46-00380261221084780] Dobson A. Bell D. (2006). Environmental citizenship. MIT Press.

[bibr47-00380261221084780] Dugmore T. I. J. Clark J. H. Bustamante J. Houghton J. A. Matharu A. S. (2017). Valorisation of biowastes for the production of green materials using chemical methods. Topics in Current Chemistry, 375(2), 46–95. doi:10.1007/s41061-017-0133-8 28374283 PMC5396386

[bibr48-00380261221084780] Escobar A. (2015). Degrowth, postdevelopment, and transitions: A preliminary conversation. Sustainability Science, 10(3), 451–462. doi:10.1007/s11625-015-0297-5

[bibr49-00380261221084780] Ferdinand M. (2019). Une écologie décoloniale. Penser l'écologie depuis le monde caribéen [Decolonial ecology. Thinking about ecology from the Caribbean world]. Édition du Seuil.

[bibr50-00380261221084780] Figueroa Sarriera H. Gray C. H. (2016). Generative justice [Special Issue]. Teknokultura: Journal of Digital Culture and Social Movements, 13(2), 361–637. doi:10.5209/rev_TK.2016.v13.n1.52388

[bibr51-00380261221084780] Fortun K. (2014). From Latour to late industrialism. HAU: Journal of Ethnographic Theory, 4(1), 309–329. doi:10.14318/hau4.1.017 PMC4800995 27011787

[bibr52-00380261221084780] Fox W. (1990). Toward a transpersonal ecology: Developing new foundations for environmentalism. State University of New York Press.

[bibr53-00380261221084780] Gatt C. (2017). An ethnography of global environmentalism: Becoming friends of the earth. Routledge.

[bibr54-00380261221084780] Ghelfi A. (2015). Worlding politics: Justice, commons and technoscience [PhD dissertation, University of Leicester].

[bibr55-00380261221084780] Ghelfi A. Papadopoulos D. (2022a). Ungovernable earth: Resurgence, translocal infrastructures, and more-than-social movements. Environmental Values. Advance online publication. doi:10.3197/096327121X16387842836968

[bibr56-00380261221084780] Ghelfi A. Papadopoulos D. (2022b). Ecological transition: What it is and how to do it. Community technoscience and green democracy. Tecnoscienza: Italian Journal of Science & Technology Studies, 13(1), 5–31.

[bibr57-00380261221084780] Gibson-Graham J. K. (2006). A postcapitalist politics. University of Minnesota Press.

[bibr58-00380261221084780] Goffey A. (2008). Abstract experience. Theory, Culture & Society, 25(4), 15–30. doi:10.1177/0263276408091980

[bibr59-00380261221084780] Gómez-Barris M. (2017). The extractive zone social ecologies and decolonial perspectives. Duke University Press.

[bibr60-00380261221084780] Graziano V. (2016). Prefigurative practices: Raw materials for a political positioning of art, leaving the avant-garde. In Malzacher F. Kampenhout E. v. Mestre L. (Eds.), Turn turtle! Reenacting the institute: Performing urgency #2. Alexander Verlag.

[bibr61-00380261221084780] Guattari F. (1995). Chaosmosis: An ethicoaesthetic paradigm. Indiana University Press.

[bibr62-00380261221084780] Gupta R. K. Thakur V. K. Matharu A. S. (2018). Editorial overview: From linear to circular economies: The importance and application of recycling and reuse. Current Opinion in Green and Sustainable Chemistry, 13, A1–A3. doi:10.1016/j.cogsc.2018.09.002

[bibr63-00380261221084780] Haas M. (2018). Tiere auf der Bühne. Eine ästhetische Ökologie der Performance [Animals on stage. An aesthetic ecology of performance]. Kulturverlag Kadmos.

[bibr64-00380261221084780] Hacking I. (1983). Representing and intervening: Introductory topics in the philosophy of natural science. Cambridge University Press.

[bibr65-00380261221084780] Hale B. Lee A. Hermans A. (2014). Clowning around with conservation: Adaptation, reparation and the new substitution problem. Environmental Values, 23(2), 181–198. doi:10.3197/096327114x13894344179202

[bibr66-00380261221084780] Halewood M. (2012). A. N. Whitehead and social theory: Tracing a culture of thought. Anthem Press.

[bibr67-00380261221084780] Haraway D. J. (1997). Modest_Witness@Second_Millennium. FemaleMan©_meets_OncoMouse™: feminism and technoscience. Routledge.

[bibr68-00380261221084780] Hartigan J. (2015). Plant publics: Multispecies relating in Spanish botanical gardens. Anthropological Quarterly, 88(2), 481–507. doi:10.1353/anq.2015.0024

[bibr69-00380261221084780] Hodson M. Evans J. Schliwa G. (2018). Conditioning experimentation: The struggle for place-based discretion in shaping urban infrastructures. Environment and Planning C: Politics and Space, 36(8), 1480–1498. doi:10.1177/2399654418765480

[bibr70-00380261221084780] Hoerl E. (2013). A thousand ecologies: The process of cyberneticization and general ecology. In Diederichsen D. Franke A. (Eds.), The whole earth: California and the disappearance of the outside (pp. 121–130). Sternberg Press.

[bibr71-00380261221084780] Hoerl E. Burton J. (Eds.) (2017). General ecology: The new ecological paradigm. Bloomsbury.

[bibr72-00380261221084780] Hopkins R. (2011). The transition companion: Making your community more resilient in uncertain times. Green Books.

[bibr73-00380261221084780] Ihde D. Selinger E. (Eds.). (2003). Chasing technoscience: Matrix for materiality. Indiana University Press.

[bibr74-00380261221084780] Iles A. (2013). Greening chemistry: Emerging epistemic political tensions in California and the United States. Public Understanding of Science, 22(4), 460–478. 23833110 10.1177/0963662511404306

[bibr75-00380261221084780] Jackson S. J. (2014). Rethinking repair. In Gillespie T. Boczkowski P. J. Foot K. A. (Eds.), Media technologies: Essays on communication, materiality, and society (pp. 221–240). MIT Press.

[bibr76-00380261221084780] Jessop P. G. (2016). The use of auxiliary substances (e.g. solvents, separation agents) should be made unnecessary wherever possible and innocuous when used. Green Chemistry, 18(9), 2577–2578. doi:10.1039/c6gc90039a

[bibr77-00380261221084780] Kallis G. (2019). Limits: Why Malthus was wrong and why environmentalists should care. Stanford University Press.

[bibr78-00380261221084780] Kingsland S. E. (2005). The evolution of American ecology, 1890–2000. Johns Hopkins University Press.

[bibr79-00380261221084780] Klein U. (2005). Technoscience avant la lettre. Perspectives on Science, 13(2), 226–266. doi:10.1162/106361405774270557

[bibr80-00380261221084780] Knorr-Cetina K. Mulkay M. J. (1983). Science observed: Perspectives on the social study of science. Sage.

[bibr81-00380261221084780] Kümmerer K. (2007). Sustainable from the very beginning: Rational design of molecules by life cycle engineering as an important approach for green pharmacy and green chemistry. Green Chemistry, 9(8), 899–907. doi:10.1039/b618298b

[bibr82-00380261221084780] Lefèvre W. Klein U. (2007). Materials in eighteenth-century science: A historical ontology. MIT Press.

[bibr83-00380261221084780] Leistert O. Schrickel I. (Eds.). (2020). Thinking the problematic: Genealogies and explorations between philosophy and the sciences. Transcript.

[bibr84-00380261221084780] Linebaugh P. (2008). The Magna Carta manifesto: Liberties and commons for all. University of California Press.

[bibr85-00380261221084780] Linebaugh P. (2010, January 8). Some principles of the commons. Counterpunch. www.counterpunch.org/2010/01/08/some-principles-of-the-commons/

[bibr86-00380261221084780] Linthorst J. A. (2010). An overview: Origins and development of green chemistry. Foundations of Chemistry, 12(1), 55–68.

[bibr87-00380261221084780] Lundy C. (2019). The call for a new earth, a new people: An untimely problem. Theory, Culture & Society, 38(2), 119–139. doi:10.1177/0263276419878246

[bibr88-00380261221084780] Macleod C. I. (2019). Expanding reproductive justice through a supportability reparative justice framework: The case of abortion in South Africa. Culture, Health & Sexuality, 21(1), 46–62. doi:10.1080/13691058.2018.1447687 29613849

[bibr89-00380261221084780] Mander J. Tauli-Corpuz V. (Eds.). (2006). Paradigm wars: Indigenous peoples’ resistance to globalization. Sierra Club.

[bibr90-00380261221084780] Mars R. Willis S. Hopkins R. (2016). The permaculture transition manual: A comprehensive guide to resilient living. New Society Publishers.

[bibr91-00380261221084780] Masciandaro G. Macci C. Peruzzi E. Ceccanti B. Doni S. (2013). Organic matter–microorganism–plant in soil bioremediation: A synergic approach. Reviews in Environmental Science and Bio/Technology, 12(4), 399–419. doi:10.1007/s11157-013-9313-3

[bibr92-00380261221084780] Masco J. (2021). The artificial world. In Papadopoulos D. Puig de la Bellacasa M. Myers N. (Eds.), Reactivating elements: Chemistry, ecology, practice (pp. 131–150). Duke University Press.

[bibr93-00380261221084780] Mathews F. (1991). The ecological self. Routledge.

[bibr94-00380261221084780] Mora-Gámez F. (2016). Reparation beyond statehood: Assembling rights restitution in post-conflict Colombia [PhD dissertation, University of Leicester].

[bibr95-00380261221084780] Naess A. (1973). The shallow and the deep, long-range ecology movement: A summary. Inquiry, 16, 95–100.

[bibr96-00380261221084780] Nixon R. (2011). Slow violence and the environmentalism of the poor. Harvard University Press.

[bibr97-00380261221084780] Nunn N. (2018). Toxic encounters, settler logics of elimination, and the future of a continent. Antipode, 50(5), 1330–1348. doi:10.1111/anti.12403

[bibr98-00380261221084780] Orr J. (2006). Panic diaries: A genealogy of panic disorder. Duke University Press.

[bibr99-00380261221084780] Ottinger G. Cohen B. R. (Eds.). (2011). Technoscience and environmental justice: Expert cultures in a grassroots movement. The MIT Press.

[bibr100-00380261221084780] P2P Foundation. (2015). Commons transition: Policy proposals for an open knowledge commons society.

[bibr101-00380261221084780] Papadopoulos D. (2018). Experimental practice: Technoscience, alterontologies, and more-than-social movements. Duke University Press.

[bibr102-00380261221084780] Papadopoulos D. Puig de la Bellacasa M. (2022). Eco-commoning in the aftermath: Sundews, mangroves and swamp insurgencies. In Urbonas N. Urbonas G. Sabolius K. (Eds.), Swamps and the new imagination: On the future of cohabitation in art, architecture, and philosophy. MIT Press.

[bibr103-00380261221084780] Papadopoulos D. Puig de la Bellacasa M. Myers N. (Eds.). (2021). Reactivating elements: Chemistry, ecology, practice. Duke University Press.

[bibr104-00380261221084780] Papadopoulos D. Puig de la Bellacasa M. Tacchetti M. (Eds.). (2022). Ecological reparation: Repair, remediation and resurgence in social and environmental conflict. Bristol University Press.

[bibr105-00380261221084780] Paxson H. Helmreich S. (2013). The perils and promises of microbial abundance: Novel natures and model ecosystems, from artisanal cheese to alien seas. Social Studies of Science, 44(2), 165–193. doi:10.1177/0306312713505003 24941610

[bibr106-00380261221084780] Perez Murcia L. E. (2013). Social policy or reparative justice? Challenges for reparations in contexts of massive displacement and related serious human rights violations. Journal of Refugee Studies, 27(2), 191–206. doi:10.1093/jrs/fet028

[bibr107-00380261221084780] Pickerill J. (2020). Making climate urbanism from the grassroots: Eco-communities, experiments and divergent temporalities. In Broto V. C. Robin E. While A. (Eds.), Climate urbanism: Towards a critical research agenda (pp. 227–242). Palgrave Macmillan.

[bibr108-00380261221084780] Pickerill J. Chatterton P. (2006). Notes towards autonomous geographies: Creation, resistance and self-management as survival tactics. Progress in Human Geography, 30(6), 730–746.

[bibr109-00380261221084780] Pignarre P. Stengers I. (2011). Capitalist sorcery: Breaking the spell. Palgrave Macmillan.

[bibr110-00380261221084780] Poliakoff M. Fitzpatrick J. M. Farren T. R. Anastas P. T. (2002). Green chemistry: Science and politics of change. Science, 297(5582), 807–810. 12161647 10.1126/science.297.5582.807

[bibr111-00380261221084780] Poliakoff M. Licence P. (2015). Supercritical fluids: Green solvents for green chemistry? Philosophical Transactions of the Royal Society A, 373, 20150018. doi:10.1098/rsta.2015.0018 PMC4650016 26574532

[bibr112-00380261221084780] Pratt M. L. (1992). Imperial eyes: Travel writing and transculturation. Routledge.

[bibr113-00380261221084780] Puig de la Bellacasa M. (2010). Ethical doings in naturecultures. Ethics, Place & Environment. A Journal of Philosophy & Geography, 13(2), 151–169.

[bibr114-00380261221084780] Puig de la Bellacasa M. (2012). ‘Nothing comes without its world’: Thinking with care. The Sociological Review, 60(2), 197–216.

[bibr115-00380261221084780] Puig de la Bellacasa M. (2015). Making time for soil: Technoscientific futurity and the pace of care. Social Studies of Science, 45(5), 691–716. doi:10.1177/0306312715599851 26630817

[bibr116-00380261221084780] Puig de la Bellacasa M. (2017). Matters of care: Speculative ethics in more than human worlds. University of Minnesota Press.

[bibr117-00380261221084780] Puig de la Bellacasa M. (2021). Embracing breakdown: Soil ecopoethics and the ambivalences of remediation. In Papadopoulos D. Puig de la Bellacasa M. Myers N. (Eds.), Reactivating Elements. Chemistry, Ecology, Practice (pp. 196–230). Durham, NC: Duke University Press.

[bibr118-00380261221084780] Ravetz J. R. (2006). The no-nonsense guide to science. New Internationalist.

[bibr119-00380261221084780] Reid H. G. Taylor B. (2010). Recovering the commons: Democracy, place, and global justice. University of Illinois Press.

[bibr120-00380261221084780] Roberts J. A. Langston N. Egan M. Frickel S. Nash L. Allen B. Vogel S. A. Rowe D. F. Daemmrich A. Murphy M. (2008). Toxic bodies/toxic environments: An interdisciplinary forum. Environmental History, 13(4), 629–703. doi:10.1093/envhis/13.4.629

[bibr121-00380261221084780] Rockström J. Steffen W. Noone K. Persson Å. Chapin F. S. Lambin E. Lenton T. M. Scheffer M. Folke C. Schellnhuber H. J. Nykvist B. de Wit C. A. Hughes T. van der Leeuw S. Rodhe H. Sörlin S. Snyder P. K. Costanza R. Svedin U. . . .Foley J. (2009). Planetary boundaries: Exploring the safe operating space for humanity. Ecology and Society, 14(2), 32–65. 10.1038/461472a 19779433

[bibr122-00380261221084780] Rosset P. a. A. M. (2017). Agroecology: Science and politics. Fernwood Publishing.

[bibr123-00380261221084780] Salvatore Engel-Di Mauro G. M. (2022). Urban food production for ecosocialism: Cultivating the CITY. Routledge.

[bibr124-00380261221084780] Sanderson K. Anastas P. T. (2011). Chemistry: It’s not easy being green. Q&A: Paul Anastas. Nature, 469(7328), 18–20. doi:10.1038/469018a 21209638

[bibr125-00380261221084780] Savransky M. (2020). Problematizing the problematic [Special Issue]. Theory, Culture & Society, 38(2), 3–159. doi:10.1177/0263276420966389

[bibr126-00380261221084780] Savransky M. (2021a). After progress: Notes for an ecology of perhaps. Ephemera: Theory & Politics in Organization, 21(1), 267–281.

[bibr127-00380261221084780] Savransky M. (2021b). Around the day in eighty worlds: Politics of the pluriverse. Duke University Press.

[bibr128-00380261221084780] Schraube E. Højholt C. (2016). Psychology and the conduct of everyday life. Routledge.

[bibr129-00380261221084780] Shattuck A. Schiavoni C. VanGelder Z. (2017). The politics of food sovereignty: Concept, practice and social movements. Routledge.

[bibr130-00380261221084780] Sheldon R. A. (2007). The E factor: Fifteen years on. Green Chemistry, 9(12), 1273–1283. doi:10.1039/b713736m

[bibr131-00380261221084780] Sheldon R. A. (2016). Green chemistry and resource efficiency: Towards a green economy. Green Chemistry, 18(11), 3180–3183. doi:10.1039/c6gc90040b

[bibr132-00380261221084780] Stengers I. (2021). Receiving the gift: Earthly events, chemical invariants, and elemental powers. In Papadopoulos D. Puig de la Bellacasa M. Myers N. (Eds.), Reactivating elements: Chemistry, ecology, practice (pp. 18–33). Duke University Press.

[bibr133-00380261221084780] Stephenson N. Papadopoulos D. (2006). Analysing everyday experience: Social research and political change. Palgrave Macmillan.

[bibr134-00380261221084780] Strathern M. (2000). Environments within: An ethnographic commentary on scale. In Flint K. Morphy H. (Eds.), Culture, landscape, and the environment: The Linacre lectures 1997 (pp. 44–71). Oxford University Press.

[bibr135-00380261221084780] Tacchetti M. Quiceno Toro N. Puig de la Bellacasa M. Papadopoulos D. (2022). Crafting ecologies of existence: More than human community making in Colombian textile craftivism. Environment and Planning E: Nature and Space. Advance online publication. doi:10.1177/25148486211030154

[bibr136-00380261221084780] Tsing A. (2012). On nonscalability: The living world is not amenable to precision-nested scales. Common Knowledge, 18(3), 505–524. doi:10.1215/0961754x-1630424

[bibr137-00380261221084780] Tsing A. (2015). The mushroom at the end of the world: On the possibility of life in capitalist ruins. Princeton University Press.

[bibr138-00380261221084780] Tuck C. O. Pérez E. Horváth I. T. Sheldon R. A. Poliakoff M. (2012). Valorization of biomass: Deriving more value from waste. Science, 337, 695–699. 22879509 10.1126/science.1218930

[bibr139-00380261221084780] Utting P. (2015). Social and solidarity economy: Beyond the fringe. Zed Books.

[bibr140-00380261221084780] van de Sande M. (2013). The prefigurative politics of Tahrir Square: An alternative perspective on the 2011 revolutions. Res Publica, 19(3), 223–239. doi:10.1007/s11158-013-9215-9

[bibr141-00380261221084780] Walker M. U. (2010). What is reparative justice? Marquette University Press.

[bibr142-00380261221084780] Wall D. (2014). The commons in history: Culture, conflict, and ecology. MIT Press.

[bibr143-00380261221084780] White R. (2016). Reparative justice, environmental crime and penalties for the powerful. Crime, Law and Social Change, 67(2), 117–132. doi:10.1007/s10611-016-9635-5

[bibr144-00380261221084780] Whitehead A. N. (1964). The concept of nature: The Tarner lectures delivered in Trinity College November 1919. Cambridge University Press.

[bibr145-00380261221084780] Whitehead A. N. (1967). Adventures of ideas. Free Press.

[bibr146-00380261221084780] Whitehead M. (2013). Editorial: Degrowth or regrowth? Environmental Values, 22(2), 141–145.

[bibr147-00380261221084780] Whyte K. (2018). Critical investigations of resilience: A brief introduction to indigenous environmental studies & sciences. Daedalus, 147(2), 136–147. doi:10.1162/DAED_a_00497

[bibr148-00380261221084780] Winner L. (1980). Do artifacts have politics? Daedalus, 109(1), 121–136.

[bibr149-00380261221084780] Woodhouse E. J. Breyman S. (2005). Green chemistry as social movement? Science, Technology & Human Values, 30(2), 199–222.

[bibr150-00380261221084780] Wyatt S. (2008). Technological determinism is dead: Long live technological determinism. In Hackett E. Amsterdamska O. Lynch M. Wajcman J. (Eds.), Handbook of science and technology studies (pp. 165–180): MIT Press.

[bibr151-00380261221084780] Zalasiewicz J. Waters C. N. Ivar do Sul J. A. Corcoran P. L. Barnosky A. D. Cearreta A. Edgeworth M. Galuszka A. Jeandel C. Leinfelder R. McNeill J. R. Steffen W. Summerhayes C. Wagreich M. Williams M. Wolfe A. P. Yonan Y. (2016). The geological cycle of plastics and their use as a stratigraphic indicator of the Anthropocene. Anthropocene, 13, 4–17. doi:10.1016/j.ancene.2016.01.002

[bibr152-00380261221084780] Zalasiewicz J. Williams M. Haywood l. Ellis M. (2011). The Anthropocene: A new epoch of geological time? [Special Issue]. Philosophical Transactions of the Royal Society, 369(1938), 833–1112. 10.1098/rsta.2010.0339 21282149

